# Ventriculoperitoneal Shunts in the Emergency Department: A Review

**DOI:** 10.7759/cureus.6857

**Published:** 2020-02-03

**Authors:** Michael Ferras, Nicholas McCauley, Trilok Stead, Latha Ganti, Bobby Desai

**Affiliations:** 1 Emergency Medicine, Ocala Regional Medical Center, University of Central Florida, Ocala, USA; 2 Emergency Medicine, Trinity Preparatory School, Winter Park, USA; 3 Emergency Medicine, Envision Physician Services, Orlando, USA; 4 Emergency Medicine, Ocala Regional Medical Center, University of Central Florida College of Medicine, Ocala, USA

**Keywords:** ventriculoperitoneal shunts

## Abstract

In this paper, we review the indications, complications, and pitfalls associated with ventriculoperitoneal (VP) shunts. As most VP shunt problems initially present to the emergency department, it is important for emergency physicians to be well-versed in managing them. In the article, the possible reasons for shunt failure are explored and summarized using an infographic. We also examine potential clinical presentations of VP shunt failure.

## Introduction and background

Emergency department physicians usually see a large number of patients with medical maladies managed by the aid of instrumentation or hardware such as a ventriculoperitoneal (VP) shunt. While patients have shunts placed for multiple reasons, it is important for emergency service providers to know how to evaluate, troubleshoot, and treat those with VP shunt complications. An estimated 30,000 VP shunt procedures are performed yearly in the United States [1]. The chances of encountering a patient with VP shunt complications in the emergency department are high. Emergency service providers must be deft at obtaining a good history and physical, as well as evaluating shunts for complications. While shunt insertion is a common neurosurgical procedure, revision rates in the first year alone can be as high as 50% in pediatric patients, whereas complication rates in adults have been reported to range between 17-33% annually [2,3]. About 4% of VP shunts will fail within two years of implantation, with a 98% failure rate over 10 years [1,4]. Shunt-related admissions to inpatient services carry an average length of stay of 8.4 days, with nearly 50% of patients requiring a stay of five or more days [5]. The mortality rate from shunt malfunction has been reported to be between 1-2.7% [5,6]. These figures are a significant source of concern and have to be borne in mind by physicians when encountering patients with shunt complications in the emergency department.

## Review

Indication for VP shunt: hydrocephalus

Hydrocephalus is defined as the abnormal or excessive accumulation of cerebrospinal fluid (CSF) around the brain [7]. The ventricular system of the human brain consists of four ventricles, lined with ependymal cells and filled with CSF. This fluid is formed at a rate of 0.35 ml/min, totaling approximately 500 ml daily in an average adult [8]. It occupies a space of 150 ml in a healthy adult patient. [8]. Communicating hydrocephalus occurs when this fluid is unable to be reabsorbed into the body, while obstructive hydrocephalus refers to a physical blockage within the ventricles of the brain [1]. The normal flow of CSF runs from the lateral ventricle to the third ventricle through the foramen of Monro. The third ventricle and fourth ventricle are connected to each other by the aqueduct of Sylvius. CSF then flows into the subarachnoid space through the foramina of Luschka and the foramen of Magendie [8]. Predictably, a blockage of the flow or failure of drainage can lead to an increase in CSF volume. The Monro-Kellie doctrine states that the human skull is a sealed container with a constant volume and, hence, an increase in the volume of fluid contained within must increase the pressure of this sealed container [8].

Hydrocephalus is not an uncommon presenting condition. The National Institute of Neurological Disorders and Stroke (NINDS) 2004 database estimates that hydrocephalus occurs in 1 out of 500 children [7]. Estimates of total incidence vary between 4-6 in every 1,000 patients [1]. The etiologies of this condition cover a wide spectrum, from infectious causes like toxoplasmosis, rubella, and cytomegalovirus to structural causes like benign masses or malignancy [7]. A host of congenital conditions including spina bifida, neural tube defects, myelomeningocele, and Dandy-Walker malformation are also associated with hydrocephalus [7].

While there are multiple forms of managing excess CSF, the placement of a VP shunt is the most common method [5]. In fact, it is one of the most common neurosurgical procedures performed in general. This is in part because the placement of a VP shunt has been shown to have the fewest complications compared to other interventions such as shunting to the atria or lung space, or more invasive surgical interventions like ventriculostomy [6,8,9]. The shunt consists of four major components: a proximal catheter, a one-way valve, a reservoir, and a distal catheter. The proximal catheter is inserted into the ventricle in the parietal occipital area and is connected to a valve just under the skin on the outside of the skull [1]. Most shunts have valves that continuously drain CSF when the ventricular pressure is greater than 10 mmHg [1]. The valve and reservoir are typically housed together just under the scalp and are easily palpated. The distal catheter is then run down into the peritoneal cavity beneath the skin, traveling down the neck and chest wall, ending in the peritoneal cavity [1].

Presentation of VP shunt infection and malfunction

The symptoms that patients with VP shunt-related pathology present with can be frustratingly vague. The chief complaints are listed below (Table 1).

**Table 1:** Usual complaints related to shunt malfunction

Symptom
Headache
Nausea, vomiting
Abdominal pain
Lethargy
Decreased intellectual performance
Ataxia
Coma
Autonomic instability
Mental status changes

Usually, complains may include headache, nausea and vomiting, irritability, somnolence, fevers, and altered mental status [9]. Patients experiencing a more severe increase in intracranial pressure (ICP) may present with bulging fontanelles, or bradycardia, while more chronic, gradual pathologies may include increasing head circumference and change in behavior or feeding patterns in pediatric patients [10]. Serious findings related to increasing cranial size in pediatric patients, apart from changes in the fontanelles, include thin-appearing or shiny scalp, palpable splitting of the cranial sutures, or changes in the percussion of the cranium, which is called Macewen’s sign (“the cracked pot sound”) [7]. More rarely, patients may present with seizures, typically accompanied by nausea or vomiting [11,12]. Increasing ICP can be difficult to diagnose early on as patients less than two weeks in age often exhibit no symptoms [7]. A caretaker report that a patient is “acting just like the last time” the patient experienced a shunt malfunction should be given appropriate weight, as the parental assessment of a change in condition may be at least as reliable as that made by a primary care physician familiar with the patient [9,11]. Likewise, adult patient presentations can be as broad as changes in personality, cognition, or memory loss [7]. Thus, it is critical to maintain a high degree of clinical suspicion when dealing with this population.

VP shunt failure

**Figure 1:**
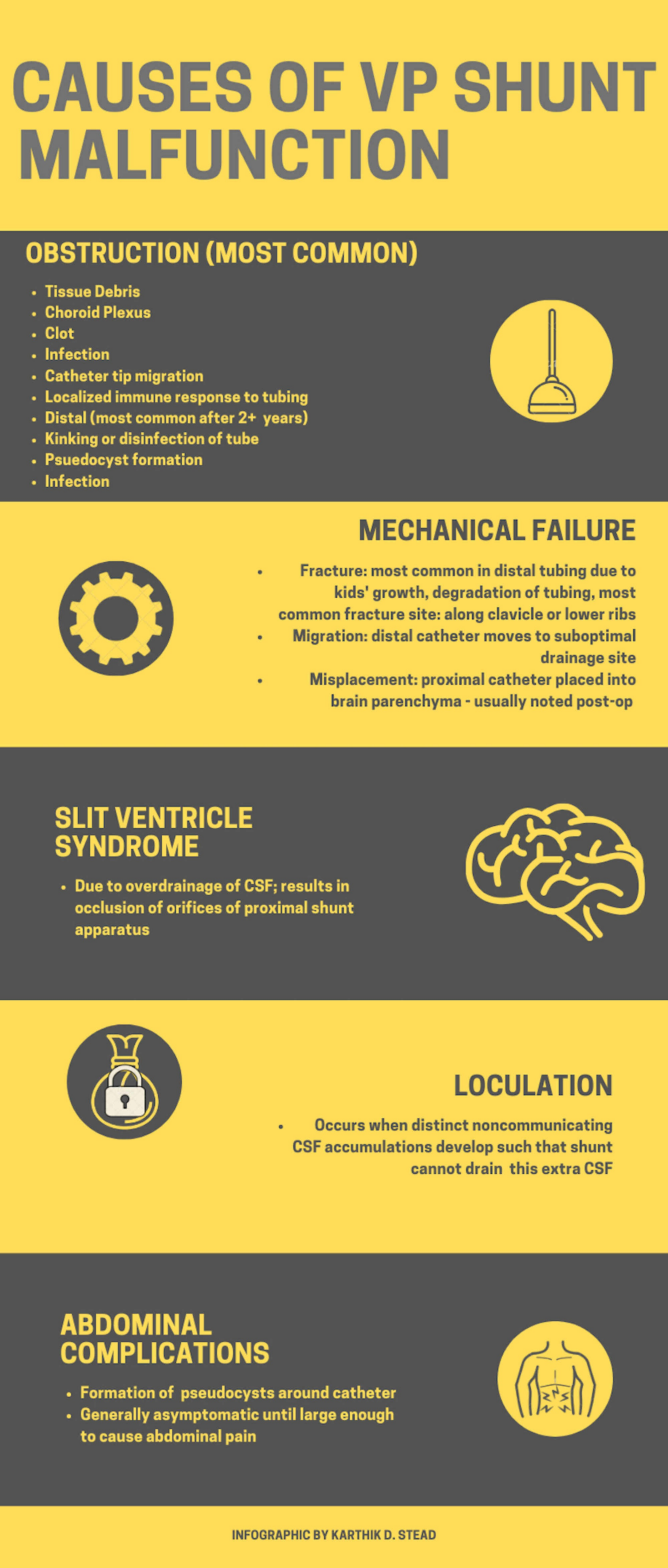
Causes of ventriculoperitoneal shunt malfunction VP: ventriculoperitoneal

Shunts can fail for a multitude of reasons that generally fall into three different categories: infection, mechanical failure, and functional failure, with mechanical failure being the most common in the United States (Figure 1).

The danger of shunt failure lies in the fact that an excess of CSF may accumulate rapidly and lead to increased ICP. In severe cases of rapid CSF accumulation in which impending herniation is suspected, emergency measures to reduce ICP must be implemented. These may include intubation and hyperventilation, mannitol, hypertonic saline, diuretics such as furosemide or acetazolamide, or surgical decompression. These interventions should be undertaken in parallel with an immediate consult to neurosurgery [6,13].

Mechanical failure can further be classified into obstruction, interruption, or malpositioning. An obstruction can form at any time after shunt placement, and the proximal catheter is the most common site, with choroid plexus or ependymal tissue entering into the catheter [14]. The other common places of obstruction are at the valve, followed by the distal catheter. Abdominal adhesions or peritoneal loculations can form obstructions distally. Fractures can also lead to shunt failure, with the most common sites of fracture being the clavicle and lower ribs. These classically occur with older catheters, but breakage may occur at any time within a shunt lifespan [6]. Commonly, fractures in the head and neck region may be seen on the clinical exam due to the extravasation of CSF under the skin. Disconnections without fracture can also occur and typically happen shortly after surgery. In growing children, migration of the catheter is also a possibility. With increasing patient height and weight, both the proximal and distal catheter can move out of position, causing resumption of symptoms and the need for shunt revision. Almost all VP shunts with mechanical failure will need neurosurgical intervention and possibly full replacement.

Infection

The yearly incidence of infection and VP shunts ranges from 5-12%, with mortality as high as 60%. The majority of these infections occur within the first two months after placement, and 90% occur within the first six months [6]. Typically, initial infections are due to pectoral skin flora such as *staph epidermidis* and *staph aureus*. However, they are uncommon and occur most often due to hematogenous spread. In very rare cases, contact with the sick can be a source of infection [7]. Fungal or parasitic infections are even rarer. In these cases, *Candida albicans* is responsible for greater than 75% of fungal infections, although other organisms such as *Aspergillus* and *Histoplasma* have also been implicated in the literature [6]. Due to the severity of illness that infections can cause, a high degree of suspicion should be maintained towards the shunt when meningitis is suspected. Since any implanted device can serve as a source of new and recurrent infection, in the case of VP shunt infection, neurosurgery should be consulted for removal and definitive management. In these cases, the expected course is that a bolt will be placed for temporary pressure monitoring after the shunt is removed by neurosurgery. Intravenous antibiotics need to be initiated early. [14]

Functional failure

Shunt functional failure can arise from a variety of clinical scenarios including overdrainage, underdrainage, and slit ventricle syndrome. Over-drainage results in a reduction in CSF volume and a corresponding drop in ICP, typically due to a confluence of factors related to shunt placement and valve malfunction. With overdrainage, patients are at risk for tearing of the bridging veins, resulting in subdural hematomas. This is typically seen within the first six months after shunt placement [14]. In slit ventricle syndrome, the pathology is similar to that of CSF over-drainage, with the appearance of small ventricles on radiography [15,16]. These patients may present with a history of headache that is aggravated when the patient is in an upright position but mitigated when the patient sleeps or is positioned laterally, in contrast with the classic pattern seen in patients with increased ICP [12].

Imaging

The most common radiologic studies in patients with concern for VP shunt-related pathology are CT of the brain, a shunt series X-ray, or a combination of the two [17]. A shunt series X-ray consists of anteroposterior (AP) and lateral views of the skull, chest, and abdomen. This is done in conjunction with a CT brain without contrast. Many institutions have protocols and order sets in place that ensure optimal imaging of the ventricles and shunt from origin to insertion. While radiography can be useful if acute shunt pathology is identified, some studies have suggested that the efficacy of the shunt series is variable. One study in adults showed a sensitivity of 88.6% and specificity of 62.5% [18]. The literature on pediatric evaluation of shunt series shows different values for sensitivity and specificity, at 11% and 98% respectively. However, when combined with a head CT, sensitivity rises to 57%, and specificity to 76% [18]. In the context of significant clinical suspicion of infection, negative imaging should not preclude consultation to neurosurgery for further guidance.

Collecting CSF

VP shunt tapping is a controversial subject even within the neurosurgical literature due to concerns regarding infection and morbidity and mortality associated with it [19]. In the instance of communicating hydrocephalus, a lumbar puncture is preferred due to the lower risk of infection and the ability to obtain the same information as from VP shunt tapping [2]. In the case of a rapidly deteriorating patient, initial efforts to lower ICP should be made using hyperventilation, mannitol, and/or 3% hypertonic saline. However, if these efforts are unsuccessful and the patient remains hemodynamically unstable, a tap of the shunt can be performed to remove CSF. Although actual infection caused by VP shunt tapping is low (<1%), employing a sterile technique is paramount [19]. It is recommended to avoid shaving the area, as it may irritate the skin and increase the risk of infection or contamination. Viable alternatives are to use a sterile lubricant to control the hair, or to use scissors to gently reduce the hair length [12]. Using a butterfly needle, IV tubing, and a syringe at a 30-degree angle, it is recommended to gently puncture the reservoir under the skin and withdraw fluid as needed. The manometer from a lumbar puncture kit may be used to measure shunt pressure. The manometer is held next to the ear when the pressures are read, with the patient positioned in a lateral decubitus position. In this arrangement, normal pressure is thought to be 8-12 mmHg [8].

## Conclusions

VP shunts are something that emergency service providers encounter quite frequently. Multiple studies show that these are fraught with complications. Hence, an emergency provider needs to be adept at diagnosing and treating acute VP shunt complications. It is imperative for emergency service providers to start with a good history and physical. This should be followed by necessary imaging procedures, and transfer to neurosurgery if required.
